# Effect of anti retroviral therapy on mucocutaneous manifestations among HIV infected patients in a tertiary care centre in India

**DOI:** 10.1186/1471-2334-14-S3-P76

**Published:** 2014-05-27

**Authors:** N Prabhakaran, TJ Jaisankar, Abdoul Hamide, M Malathi, DM Thappa

**Affiliations:** 1 Department of Dermatology, Jawaharlal Institute of Post Graduate Medical Education and Research (JIPMER), Puducherry, India

## Background

A panorama of mucocutaneous manifestations is produced by HIV infection which correlates with the degree of immunodeficiency. Viral, bacterial and fungal infections, as well as inflammatory skin diseases, have all been reported to increase as CD4 T cells are depleted. Since the introduction of HAART, there has been a dramatic decrease in the incidence of HIV associated dermatoses. However, initiation of ART itself causes various cutaneous adverse effects. Our goal was to study the various mucocutaneous manifestations encountered in treatment naïve HIV infected and those on ART.

## Methods

This study involved a cross sectional assessment of 85 HIV seropositive patients attending STD and ART clinic. Of which 16 patients were not on HAART and the rest 69 were on HAART.

## Results

The mean baseline CD4 count of those not on HAART was 571.94±278.44 and mean baseline CD4 count of those on HAART was 281.88± 231.02 and the difference was significant (*p*<0.0001). Six months post HAART, the mean CD4 count had significantly increased to 515.44± 245.54 (*p* <0.001). The incidence of mucocutaneous manifestations were similar in both the groups (*p*=0.4891). The most common infections in those not on HAART were fungal (25%), viral (12.5%), bacterial (6%). And among those on HAART were fungal (43%), viral(25.5%) and bacterial(2.8%). The various adverse cutaneous reactions to HAART observed in our study were lipodystrophy (14%), maculopapular rash(7.2%) lichenoid eruptions(5.2%).

## Conclusion

These findings indicate that HAART often reduces the incidence of infectious skin diseases in patients with HIV, but can itself be the cause of drug eruptions.

